# Common bacteria in sputum or gastric lavage of patients presenting with signs and symptoms of lower respiratory tract infections

**DOI:** 10.11604/pamj.2021.38.383.26333

**Published:** 2021-04-20

**Authors:** Oliver Deberu, Bernard Nkrumah, Augustina Angelina Sylverken, David Sambian, Godfred Acheampong, John Amuasi, Azure Stebleson, Daron Agboyie, Monica Yenbaree, Sylvester Mensah, Abaifa Dombadoh, Dorcas Ohui Owusu, Abass Abdul-Karim, Michael Owusu

**Affiliations:** 1 Tamale Public Health Laboratory, Tamale, Ghana,; 2 Centre for Health Systems Strengthening, Kumasi, Ghana,; 3 African Field Epidemiology Network, Accra, Ghana,; 4 Department of Theoretical and Applied Biology, Kwame Nkrumah University of Science and Technology, Kumasi, Ghana,; 5 Department of Global Health, School of Public Health, Kwame Nkrumah University of Science and Technology, Kumasi, Ghana,; 6 Garden City University College, Kumasi, Ghana,; 7 Department of Medical Diagnostics, Kwame Nkrumah University of Science and Technology, Kumasi, Ghana

**Keywords:** Sputum, pathogens, tuberculosis, culture, lower respiratory tract, Tamale Public Health Laboratory, infection

## Abstract

**Introduction:**

lower respiratory tract infections (LRTIs) are infections involving the trachea, primary bronchi and lungs. People with LRTIs typically experience coughs as the primary symptoms; however, shortness of breath, weakness, fever and fatigue may be coupled with the cough. It is common among the aged, children under five and the immune-suppressed. Persons with symptoms suggestive of pulmonary tuberculosis (TB) may have tuberculosis, other respiratory tract infection or co-infection of tuberculosis and other respiratory pathogens. This study aimed to identify the presence of pathogens in sputum of suspected tuberculosis cases and their antimicrobial resistance patterns.

**Methods:**

this was a retrospective study conducted from September 2018 to November 2019 at Tamale Public Health Laboratory. Sputum or gastric lavage samples were collected from persons with suspected clinical presentations of TB and/or LRTI. These samples were cultured using standard microbiological protocols and antimicrobial susceptibility test performed on the positive cultures by Kirby-Bauer disc diffusion method. Molecular identification of M. tuberculosis was performed on all the suspected TB cases using GeneXpert mycobacterium tuberculosis/rifampin (MTB/RIF) assay.

**Results:**

during the study period, there were 264 cases of which 49.2% were males and 50.8% were females. Positive cases for culture were 47.3%. Out of the 264 cases, 186 (70.5%) were suspected TB with 51.6% being positive for culture, 6.5% positive for M. tuberculosis (GeneXpert confirmed) and 3.8% co-infection of TB with other bacteria pathogens. Klebsiella spp. (35/125; 28%) and Pseudomonas spp. (19/125; 15.2%) were the most predominant pathogens isolated. There was no significant difference in detection of bacteria in males and females (p=0.89), however individuals with suspected TB were significantly infected with other bacterial species than the unsuspected individuals (p=0.03). Almost all the isolates showed high susceptibility towards carbapenem (meropenem) and high resistance towards the third generation cephalosporins (cefotaxime and ceftriaxone).

**Conclusion:**

this study highlights the need to test individuals with classical symptoms of LRTIs for other bacterial infections other than TB only. Sputum culture is recommended for all suspected tuberculosis cases to provide accurate laboratory diagnosis to LRTIs and mitigate unnecessary use of antimicrobials.

## Introduction

Lower respiratory tract infections (LRTIs) are infections which affect the trachea, primary bronchi and the lungs [1]. The most common clinical presentation with LRTIs is coughing; however, shortness of breath, weakness, fever and fatigue may also be present [2]. Lower respiratory tract infections is known to be one of the most important unrestricted health problems and a principal cause of morbidity and mortality in several developing countries [3]. It is a worldwide predicament accounting for more than 5 million deaths each year and occurs in both community and health care settings [4]. In Africa, LRTIs are one of the most common causes of death [5]. In developing countries, the condition is more complex and difficult to manage due to limited capacity to identify the causative agents and inappropriate administration of antibiotics [5].

Lower respiratory tract infection is common among the aged, children under five and the immune-suppressed [6]. Persons with symptoms suggestive of LRTIs may have tuberculosis (TB) and/or other bacterial and viral infections [7-9]. Over the years, most severe cases of pneumonia have been associated with *M. tuberculosis*, with little information on other relevant bacterial pathogens [10,11]. Some common pathogens causing LRTIs other than *M. tuberculosis* include *Streptococcus pneumoniae, Haemophilus influenzae, Klebsiella pneumonia* and *Pseudomonas aeruginosa* [12]. Correct identification of these pathogens and good antimicrobial susceptibility tests provide great guidance to clinicians for better management of the infection [13]. However, the absence of these practices create avenue for misdiagnosis and misuse of antibiotics leading to antibiotic resistance.

Antimicrobials should only be prescribed based on a credible culture and sensitivity laboratory test results [14]. Antimicrobial resistance is promoted especially in remote areas where laboratory testing is barely done [15].

As part of capacity building for microbiological investigations, the Tamale Public Health Laboratory (TPHL) received logistics support and training in performing microbiological cultures on various samples including sputum and gastric lavage. The capacity building programme was supported by the United States Centers of Disease Control and Prevention (US CDC) and implemented by the Centre for Health Systems Strengthening (CfHSS; Ghana) and the Association of Public Health Laboratories (APHL; USA). Prior to the programme implementation, microbiological cultures were not performed at the public health laboratory. This report describes the various microbiological pathogens identified in sputum and gastric lavage as a result of the capacity building programme.

## Methods

**Study area:** the study was conducted at the Tamale Public Health Laboratory (TPHL). This laboratory serves as a meningitis referral laboratory for the five northern regions and the upper part of Brong Ahafo and Volta regions of Ghana. Tamale is located in the centre of the northern region with an approximated land size of 646.90180 sqkm with a population of 233,252 inhabitants [16]. Tamale Public Health Laboratory is located within the premises of the Tamale Teaching Hospital which is the third largest teaching hospital in Ghana after the Korle Bu Teaching Hospital and Komfo Anokye Teaching Hospital.

**Study design and study population:** this was a retrospective cross-sectional study conducted from September, 2018 to November, 2019 as part of CfHSS objective to strengthen laboratories core capacities in disease diagnosis in Ghana. The study population comprised patients of all ages and gender with suspected clinical presentations of tuberculosis and/or lower respiratory tract infection. Participants with classical symptoms of tuberculosis were termed “suspected TB” subjects and those with other general symptoms of respiratory disease were termed “unsuspected TB” subjects. Suspected TB subjects presented with chest pains, difficulty breathing, cough (with or without blood) that lasts more than 3 weeks, night sweats, chills, fever and weight loss. Unsuspected TB patients presented with symptoms including chronic obstructive pulmonary tuberculosis, bronchopneumonia, bronchitis, lung abscess, pulmonary edema, congestive cardiac failure and other respiratory conditions unrelated to tuberculosis.

**Data collection:** data for this study comprised archived records of sputum and gastric lavage retrieved from the data department of TPHL. Patients´ biodata such as age, gender, clinical presentation of TB and/or LRTI and type of specimen were reviewed and collected for analysis. Incomplete data were excluded.

**Specimen collection:** patients produced the sputum specimens themselves. Individuals classified as “suspected TB” patients produced two sputum specimens into two separate containers (50 ml wide-neck). For one specimen, confirmation of *M. tuberculosis* by GeneXpert was done. For the second specimen, isolation and identification of other organisms by microbiological culture was conducted. “Unsuspected TB” patients only submitted one specimen for bacteriological culture investigation. Gastric lavage aspiration was taken from individuals, typically infants, who could not produce sputum and the aspiration was done by trained clinicians, under sterile condition. All clinical specimens from “suspected TB” subjects were tested for tuberculosis and other bacteria using GeneXpert analysis and bacteriological cultures, respectively. Samples from “Unsuspected TB” subjects were only tested for other bacterial pathogens (excluding TB) using conventional bacteriological investigations.

**Specimen processing by microbiological culture:** bacteriological investigation was performed on sputum and gastric lavage specimens by inoculated specimen on chocolate agar (CA), blood agar (BA) and MacConkey agar using sterile disposable loop (10 μl). MacConkey and BA were incubated overnight under aerobic conditions at 35-37°C while CA plates were incubated in candle jar for microaerophilic condition at the same temperature. The plates were observed for bacterial growth. Morphological characteristics, lactose fermentation, haemolysis on BA, gram staining and biochemical reactions were used to give presumptive identification. All gram-negative bacteria were biochemically confirmed using analytical profile index (API) 20E or 20NE (Biomerieux, France) following all standard microbiological procedures. All gram-positive bacteria (typically Staphylococci and Streptococci) were identified using colony morphology, hemolysis on BA, gram reactions, catalase and coagulase tests.

**Antimicrobial susceptibility testing:** antimicrobial susceptibility was performed using Kirby-Bauer disc diffusion method. Pure growth of bacteria less than 24 hours was used to set-up the antimicrobial susceptibility tests. Gram-negative bacteria susceptibility to amoxiclav (amoxicillin and clavulanic acid; 20/10 μg), ceftriaxone (30 μg), azithromycin (15 μg), amikacin (30 μg), meropenem (10 μg), trimethoprim/sulfamethoxazole (1.25/23.75 μg), ciprofloxacin (5 μg), gentamicin (10 μg), ceftazidime (30 μg) and cefotaxime (30 μg) were tested on Mueller-Hinton agar (BD, USA). The antibiotics were selected based on the clinical and laboratory standards institute (CLSI) guidelines [17]. The sizes of zone of inhibition (mm) were determined if bacteria were resistant or susceptible to different antibiotics based on CLSI guidelines. Antibiotic discs used for the susceptibility testing were quality controlled weekly using appropriate American type culture collection (ATCC) strains. Also, quality control testing was performed anytime an antibiotic with a new lot number was used in the process. Zone diameters (mm) were recorded in the quality control (QC) log following the CLSI guidelines.

**Sample processing by GeneXpert MTB/RIF:** for “suspected TB” patients, 4 ml of the reaction buffer was added to 2 ml of the sputum or gastric lavage sample, vortexed and incubated at room temperature for 15 minutes. Two microlitres of the homogenised sample was loaded into the cartridge and subsequently inserted into the GeneXpert machine after barcode registry. Results was ready after 90 minutes. The principle of the GeneXpert MTB/RIF assay is based on the binding of specific primers to complimentary sequence on the DNA of *Mycobacterium tuberculosis* if present.

**Ethical approval:** permission was granted from the head of TPHL before collection of this data. The main study protocol was reviewed and approved by the Ethical Review Committee (ERC) of the Ghana Health Service (approval number: GHS-ERC008/03/20).

**Data analysis:** data were entered into Microsoft Excel and analyzed using STATA version 12 (Stata Corp, USA) and GraphPad Prism version 6.01 (San Diego, USA). Descriptive statistics such as proportions and frequencies were used to describe the variables. Differences between discrete variables were analyzed using chi-square. P<0.05 was considered statistically significant.

## Results

**General characteristics of the study population:** during the study period, a total number of 264 samples were collected: 260 (98.5%) sputum and 4 (1.5%) gastric lavage. Samples collected from females (154; 50.8%) were slightly more than that of males. More than half (59.5%) of the samples collected were from individuals aged over 41 years (Table 1). Majority of the individuals recorded had suspected cases of TB (186; 70.5%).

**Table 1:** demographic characteristics of participants

Variable	Frequency (n = 264), n (%)		
	Positive cultures	Negative cultures	p-value
**Sex**			
Male	61 (23.1)	69 (26.1)	0.89
Female	64 (24.2)	70 (26.5)	
**Age (years)**			
≤20	13 (4.9)	14 (5.3)	0.58
21 - 30	15 (5.7)	14 (5.3)	
31 - 40	14 (5.3)	21 (8.0)	
41+	83 (31.4)	74 (28.0)	
**Suspected TB**			
Yes	96 (36.4)	90 (34.1)	0.03
No	29 (11.0)	49 (18.6)	
**Sample type**			
Sputum	124 (47.0)	136 (51.5)	0.37
Gastric lavage	1 (0.4)	3 (1.1)	

**Figure 1:**
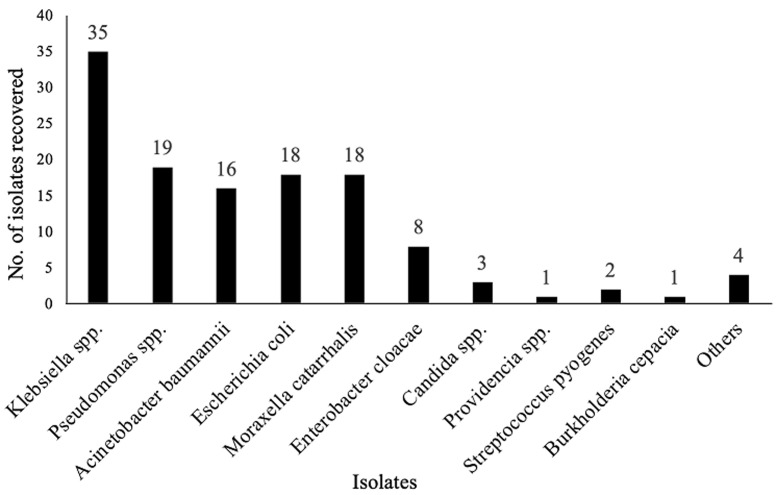
distribution of isolates recovered from sputum

**Identification of bacteria from sputum and gastric lavage:** of 264 samples collected and analyzed, 125 (47.3%) isolates were recovered from both sputum and gastric lavage. Of the 125 bacterial organisms identified, 96 (35.4%) were from suspected TB patients and 29 (11.0%) were from unsuspected TB patients. Gastric lavage produced only one (0.8%) organism (*Candida spp*.) from an unsuspected TB individual. Sputum analysis revealed 13 different species of bacteria and one fungal organism (Figure 1). The most predominant bacteria isolated was *Klebsiella spp*. (35/125; 28%). Other isolates include *Pseudomonas spp*. (19/125; 15.2%), *Acinetobacter baumannii* (16/125; 12.8%), *Escherichia coli* (18/125; 14.4%) and *Moraxella catarrhalis* (18/125; 14.4%). Only one isolate each of the bacteria *Pasteurella pneumotropica, Proteus spp*., *Staphylococcus spp*. and *Raoultella ornithinolytica* were isolated, hence this was combined and categorised as ‘others´ (Figure 1).

**Effects of sociodemographic characteristics on LRTIs:** generally, females (24.2%) were mostly infected with organisms causing lower respiratory tract infection than males (23.1%), although it was not statistically significant. A total number of 78 individuals with unsuspected symptoms of TB were not tested for *M. tuberculosis* (Table 1). Individuals with suspected tuberculosis were significantly infected with other bacterial species than the unsuspected individuals (p=0.03). Older individuals (40 years and above) had the most isolates recovered from their sputum and gastric lavage, however, this was not statistically significant.

**Bacteria and TB coinfections:** of the 186 suspected TB patients who were screened with GeneXpert, only 12 (6.45) tested positive for TB. Six individuals with confirmed pulmonary tuberculosis infection had bacteria co-infection within their lower respiratory tract. However, among the 174 tuberculosis negative individuals, 90 (51.7%) had bacteria other than *Mycobacterium tuberculosis* from the sputum samples collected (Table 2). There was however no statistically significant difference in bacteria detection between TB positive individuals and non-TB persons (p=0.91).

**Table 2:** lower respiratory tract infections among TB positive and negative subjects

Isolates	TB positive (n = 12)	%	TB negative (n = 174)	%	Total (n = 186)	%
*Klebsiella* spp.	0	0	27	15.5	27	14.5
*Pseudomonas spp*.	2	17	13	7.5	15	8.1
*Acinetobacter baumannii*	1	8	13	7.5	14	7.5
*Escherichia coli*	1	8	11	6.3	12	6.5
*Moraxella catarrhalis*	1	8	13	7.5	14	7.5
*Enterobacter cloacae*	0	0	5	2.9	5	2.7
*Candida* spp.	0	0	2	1.1	2	1.1
*Providencia spp*.	0	0	1	0.6	1	0.5
*Streptococcus pyogenes*	0	0	1	0.6	1	0.5
*Burkholderia cepacia*	0	0	1	0.6	1	0.5
*Others*	1	8	3	1.7	4	2.2
Total	6	50.0	90	51.7	96	51.6

**Figure 2:**
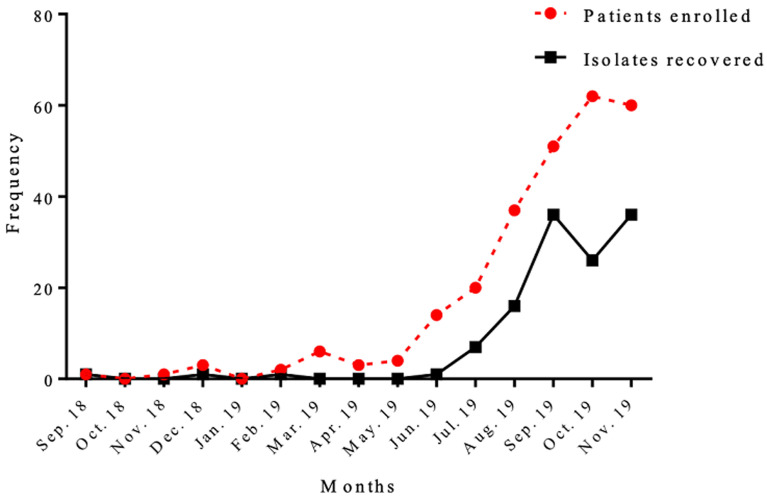
monthly trend of patients enrolled and isolates recovered throughout the study period

**Distribution of samples and isolates recovered by months:** this study was conducted within a period of 15 months. Cases of respiratory infections was low for the first 9 months from inception of the study, until June, 2019, where samples saw a 3-fold increase (Figure 2). Cases of respiratory infection increased progressively with increased sample processing and isolates recovered until the end of study, although there was no statistically significant difference between samples collected and isolates identified (p=0.09).

**Antimicrobial resistance profile of most common bacteria isolated:** *Klebsiella spp*. was highly resistant to third generation cephalosporins especially ceftriaxone (14/35; 40%). Meropenem and Amikacin showed complete efficacy against all the *Klebsiella*tested. Gentamicin was least effective against *Pseudomonas spp*. but not *Acinetobacter baumannii* (Table 3). Similar to *Klebsiella*, ceftriaxone was the antibiotic with least activity (10/16; 62.5%) against *Acinetobacter baumannii*, followed by amoxicillin/clavulanic acid (8/16; 50%). Of 18 *Escherichia coli* isolates tested, half were resistant to Gentamicin. Ceftriaxone and Amikacin were least effective against *E. coli* with rate of resistance of 61.1% each. Meropenem was totally potent against *E. coli*, but not *Acinetobacter baumannii*. Antimicrobial activities against *Moraxella catarrhalis* and *Enterobacter cloacae* were lowest in Amikacin (5/18; 27.8%) and Amoxiclav (7/8; 87.5%), respectively.

**Table 3:** rate of antimicrobial resistance among commonly isolated bacteria

	% resistance					
Antibiotics	Klebsiella spp. (n=35)	Pseudomonas spp. (n=19)	Acinetobacter baumannii (n=16)	Escherichia coli (n=18)	Moraxella catarrhalis (n=18)	Enterobacter cloacae (n=8)
GEN	22.9	26.3	0	50.0	5.6	0
CIP	11.4	5.3	25.0	38.9	22.2	12.5
CTX	25.7	15.8	43.8	22.2	-	12.5
CRO	40.0	10.5	62.5	61.1	5.6	37.5
CZM	28.6	5.3	6.3	38.9	11.1	-
AZM	11.4	5.3	-	22.2	-	12.5
AMK	0	5.3	-	5.6	27.8	-
AMC	25.7	15.8	50.0	61.1	-	87.5
SXT	11.4	10.5	6.3	22.2	22.2	37.5
MEM	0	0	6.3	0	-	-

GEN: gentamicin; CIP: ciprofloxacin; CRO; ceftriaxone; CZM: ceftazidime; AZM: azithromycin; AMK: amikacin; AMC: amoxicillin/clavulanic acid; SXT: trimethoprim/sulfamethoxazole; MEM: meropenem; CTX: cefotaxime

## Discussion

This study reports on high number of isolates (125/264; 47.3%) recovered from sputum and gastric lavage of individuals attending the Tamale Public Health Laboratory. Laboratory analysis of sputum specimens revealed 13 different bacterial species and a fungus associated with lower respiratory tract infections. Again, individuals with suspected tuberculosis were significantly infected with other bacterial species.

Lower respiratory tract infection is a leading cause of death globally and it comprises wide range of diseases such as bronchitis, pneumoniae and acute exacerbation of chronic lung diseases [18]. In developing countries, the situation is more complicated as a result of problem associated with identification of the etiological agents [19]. The present study utilized microbiological techniques to recover bacteria isolates, mainly from the sputum of patients. The total prevalence of microbial isolates from TPHL was 47.3%. Earlier studies conducted in Cape Coast Teaching Hospital in Ghana reported prevalence of 84.9% [20]. Similar reports in Nepal and India also identified high pathogen isolation rates of 43.7% and 39%, respectively, from the sputum [21]. Bronchoscopic studies have shown that 50% of patients harbour these bacteria in their lower airways and they are significant cause of exacerbations [22].

Twenty-nine isolates were recovered from patients with unsuspected cases of TB, accounting for a prevalence of 10.9%. This prevalence is low compared to studies conducted in Enugu State, Nigeria (45.2%) and Tunisia (24%) [23,24]. These differences in prevalence may be explained by differences in study designs and geographic locations. Indeed, the spread of respiratory infections varies between populations and countries, depending on disparities in geography and socio-economic conditions [25,26].

More than half of the suspected TB cases (96/186; 51.6%) were culture positive for other bacteria, resulting in the identification of 125 non-mycobacterial pathogens. GeneXpert detected *Mycobacterium tuberculosis* in twelve (6.5%) suspected cases and 6 (3.2%) had co-infection with other pathogens. The use of GeneXpert has been strongly recommended by a study in Kenya, which identified an additional 29.5% prevalent cases, missed by sputum microscopy and culture [27]. More than 90% of the global TB cases and deaths occur in the developing countries [28]. Report by Kyu and colleagues revealed that most of Asia, Eastern Europe and all of sub-Saharan Africa has higher tuberculosis burden than expected given their level of sociodemographic development [29]. Evidence has shown that not all individuals who are exposed to the *M. tuberculosis* progress to having active TB infections [30]. Studies from high burden TB environments suggest that about 20% of people maintain negative tuberculin skin tests throughout their lifespan despite repeated exposure to the mycobacteria [31].

Tuberculosis co-infection with other bacteria detected was comparatively lower than that reported by Attia and colleagues in Cambodia, with 33% of TB patients having other bacterial infections [32]. Other bacterial pathogens co-infection with TB have been described in several populations, especially, those with a high TB prevalence [33,34]. Differentiating TB from other LRTIs such as bacterial pneumonia is an important clinical challenge in these populations and this may result in poorer health outcomes [35,36]. We only included patients with sputum specimens that were tested for both mycobacterial and bacterial pathogens; yet, for majority of patients who attend hospitals in Ghana with respiratory symptoms, sputum samples are usually tested for tuberculosis alone without culture for other microbial organisms. Few *Candida spp*. were detected by culture and these could possibly be associated with pulmonary mycoses. These are rarely detected and are a major threat for immunocompromised patients [37].

Most of the non-mycobacterial pathogens were isolated from older individuals (40 years and above), similar to a study in India which showed higher prevalence of LRTI in patients over 50 years [38]. This could be due to decreased immunity in the aged. However, this is inconsistent with a study in Nigeria which reported the least prevalence in participants above 60 years. A critical look into that study revealed that there were relatively few number of individuals recruited who were above 60 years (24 patients), compared with those below 60 (141 patients) [39]. Of the thirteen different non-mycobacterial pathogens identified in this study, *Klebsiella spp*. was the most prevalent pathogen (28%), followed by *Pseudomonas spp*. (15.2%) and *Escherichia coli* (14.4%). This is consistent with studies conducted elsewhere in Kathmandu (Nepal) and Abeokuta (Nigeria) with *Klebsiella, Pseudomonas* and *E. coli* as predominant causes of LRTIs [39,40]. Several Indian studies have also reported higher prevalence of *Klebsiella pneumonia* as the cause of LRTIs [38,41]. Although these bacteria are normal flora of the gut, their presence are mostly associated with infections of the urogenital tract and peritoneum, with occasional infections at distant loci after bacteremia [42]. However, their involvement in pulmonary infections are rarely implicated, inducing bronchopneumoniae with interstitial infiltration of mononuclear cells leading to more rapid deterioration of lung function [42,43].

Antibiotic susceptibility tests indicated that the isolates were resistant to one or more antibiotics. *Klebsiella spp*. showed higher resistance towards the third generation cephalosporins (cefotaxime; 25.7% and ceftriaxone; 40.0%) but was completely susceptible to meropenem and amikacin. Again, Gentamicin was least effective against *Pseudomonas spp*. and *E. coli* but not *Acinetobacter baumannii*. These observations are worrisome and pose serious public health problems, as documented in other studies [44,45]. Although *Klebsiella, Pseudomonas* and *E. coli* have been shown to be resistant to many antimicrobials in this study and others [38,39], their mechanism of resistance was not established. Most common mechanisms found to be associated with gram-negative bacteria resistance to antibiotics include acquisition of genes encoding extended spectrum β-lactamases (ESBL), metallo-β-lactamase (MBL) and ampC β-lactamase production [38].

One limitation of the study was the small sample size attained during the study period, especially from the gastric lavage. Interpretation of data should therefore be done with caution. Another limitation was the inability to test for the presence of extended spectrum beta-lactamases (ESBL) phenotypes in the bacterial isolates that were resistant to third generation cephalosporins (cefotaxime and ceftriaxone). This was due to logistical constraints.

## Conclusion

Lower respiratory tract infections pose significant public health threat globally. This study has shown the need to test individuals with classical symptoms of LRTIs for other bacterial infections other than TB only. Blind antibiotic treatments which put the lives of patients at risk should be discouraged. This study strongly recommends that, microbiological culture and sensitivity should be done for all suspected TB cases. Culture and antimicrobial susceptibility tests should be performed before administration of any antibiotics. Surveillance and quality systems should be strengthened, to aid in reshaping current concepts toward control of LRTIs as a public health problem.

**Funding:** this work was supported by EDCTP2 programme and the Centre for Health Systems Strengthening (CfHSS), Kumasi, Ghana.

### What is known about this topic

- Lower respiratory tract infections (LRTIs) are one of the most common causes of death in Africa;
- About a third of the world´s population is infected with Mycobacterium tuberculosis with about 95% of cases arising from sub-Sahara Africa.

### What this study adds

- This study reports significant numbers of pathogenic bacteria recovered from sputum and gastric lavage of individuals in Northern Ghana;
- This study has shown the need to test individuals with clinical presentations of LRTIs for other bacterial infections other than tuberculosis only.
